# Cluster-Randomized Trial of a Multilevel Parish-Based Intervention to Improve Mental Health Literacy and Reduce Stigma in Hispanic Communities

**DOI:** 10.21203/rs.3.rs-7017764/v1

**Published:** 2025-10-26

**Authors:** Eunice C. Wong, Mario O. Martinez, Bing Han, Rachana Seelam, Kathryn P. Derose

**Affiliations:** RAND Corporation; Roman Catholic Diocese of San Bernardino; Kaiser Permanente Research and Evaluation; RAND Corporation; University of Massachusetts

**Keywords:** Stigma, Mental Health Literacy, Faith Community, Hispanic, Parishes, Contact-Based Education

## Abstract

**Purpose:**

Greater risk for stigma and low mental health literacy have been attributed to the longstanding mental health treatment disparities experienced by the U.S. Hispanic population. Limited research has focused on developing and evaluating effective interventions targeting these disparities. We examined the effectiveness of a multilevel, multicomponent, parish-based intervention involving a partnership between the Diocese of San Bernardino and the National Alliance on Mental Illness in which contact-based education was delivered to Hispanic church attendees.

**Methods:**

This cluster-randomized trial enrolled 1,715 participants who were recruited from 14 parishes that were randomly assigned to the intervention (N = 911) or wait-list condition (N = 804). Baseline and 12-month surveys assessed outcomes related to mental health stigma and literacy, support provided to individuals with mental health problems, mental health service use, and psychological distress.

**Results:**

Intent-to-treat analyses indicated no significant intervention effects. Only 45% (N = 412) of respondents within the intervention parishes reported exposure to at least one intervention activity. As-treated analyses showed that compared to control parish participants, intervention parish participants who were exposed were significantly less likely to social distance from and more likely to provide support to individuals with mental illness and had greater decreases in rates of serious psychological distress.

**Conclusions:**

This study demonstrated when exposed to a multilevel, multicomponent stigma intervention within a faith-based congregational setting long-term positive outcomes could be achieved. Findings reinforce how partnerships with faith communities can play an integral role in addressing stigma and unmet mental health needs.

**ClinicalTrials.gov Identifier:**

NCT03631745

## INTRODUCTION

The U.S. Hispanic population continues to be disproportionately affected by high levels of untreated mental illness [1,2], delayed treatment initiation[3], and treatment dropout [4].^[1]^ For instance, only 36% of U.S. Hispanic adults with a mental illness received mental health services compared to 52% of White adults [5]. Stigma and mental health literacy have been identified as key contributors to these intransigent disparities [6]. Higher levels of stigma have been documented among Hispanic individuals who have been shown to hold more negative attitudes toward people with mental illness as well as greater feelings of shame and embarrassment about having a mental illness relative to non-Hispanic White individuals [7–9]. Relatedly, Hispanic individuals who express fears of being stigmatized and discriminated against because of a mental illness are more reluctant to both acknowledge having a mental health problem and to utilize mental health services [10,11].

Mental health literacy, defined as the “knowledge and beliefs about mental disorders which aid in their recognition, management or prevention” is essential for identifying mental illness, recognizing the need for treatment, and ultimately obtaining treatment [12]. Hispanic individuals with serious mental illness are less likely to perceive a need for treatment relative to non-Hispanic individuals [13]. Moreover, a substantial proportion of Hispanic individuals have also endorsed mental health knowledge-related barriers, such as not knowing where to obtain services, beliefs that treatment is ineffective, and fear that psychotropic medications are addictive [14–16].

Despite higher risks for mental health-related stigma and low mental health literacy among Hispanic individuals, research focused on developing and evaluating interventions targeting these disparities is limited [6]. A recent review identified only seven studies (four employed the same depression fotonovela brochure) that evaluated mental health literacy and stigma interventions with Hispanic individuals and found promising results for mental health literacy outcomes but mixed findings for stigma outcomes [6]. Further, the review noted a number of methodological limitations that are critical to address in order to advance evidence-based mental health literacy and stigma interventions for Hispanic populations. For example, of the seven studies identified, only two were randomized control trials, most had small sample sizes, and all assessed short-term outcomes of less than six months.

A meta-review of stigma interventions among the broader population revealed limited evidence for the longer-term impact of stigma interventions though there was some indication that multi-component contact interventions such as contact-based education may be effective in decreasing stigma longitudinally [17]. Contact-based education involves contact with individuals who provide not only mental health education but their own personal stories of recovery from mental illness [18]. Though one of the most widely researched interventions, contact-based education interventions have been primarily limited to certain segments of the population (e.g., healthcare professionals, students); thus, the National Academy of Sciences has recommended that future targets of stigma reduction research and intervention expand to other audiences that are integral to reach in order to create more supportive environments such as faith-based leaders and family and friends of individuals with mental illness [18].

Faith communities can play a critical role as gatekeepers by facilitating the early detection of mental illness, shaping social norms and stigma around mental illness and treatment, and establishing linkages to mental health care as documented in a recent review on partnerships between the faith community and mental health sector [19]. Given that a substantial proportion of the U.S. Hispanic population turn to faith communities when confronted with mental health problems, faith communities can play a vital role in reducing mental health disparities [20,21]. In fact, faith communities serving racial-ethnic minoritized communities have been found to be more willing to refer and collaborate with the mental health sector perhaps because of their more extensive involvement in the delivery of social service programs, crisis intervention, and counseling with individuals with mental illness [22,23]. However, faith communities report major obstacles to collaborating with the mental health sector including the lack of mental health training, staffing, and resources [24,25].

Partnerships that leverage the collective resources of faith communities and community-based mental health organizations may be a promising means to targeting sources of mental health disparities among Hispanic individuals [26,27]. Such partnerships may provide a viable and sustainable approach to disseminating mental health literacy and stigma reduction programs within faith communities and in establishing pathways to mental health care. Thus, we examined the effectiveness of AMEN (Alma, Mente, Espíritu, y Nuestra Communidad/Soul, Mind, Spirit, and Our Community), a multilevel, multicomponent, parish-based intervention involving a partnership between the RAND, the National Alliance on Mental Illness (NAMI) and the Roman Catholic Diocese of San Bernardino (which presides over parishes in California’s Riverside and San Bernardino counties, respectively ranked as 6^th^ and 8^th^ of U.S. counties with the largest Hispanic population) [28]. NAMI (nami.org), the largest grassroots mental health organization in the U.S., has developed a host of programs tailored to the different needs and segments of the community affected by mental illness, including programs uniquely designed to address culturally diverse and faith-based communities. A defining feature of NAMI is that their programs are delivered by individuals and their family members who have lived experience recovering from a mental illness, which essentially constitutes contact-based education, a type of intervention that has been shown to sustain longer term reductions in stigma [17]. We conducted a cluster-randomized trial with baseline and 12-month follow-up data to evaluate whether predominantly Hispanic parishes receiving AMEN intervention activities would experience decreases in stigma, increases in mental health literacy, and improvements in support provision, access to mental health services, and psychological distress.

## METHODS

This study entailed a collaboration between researchers at RAND, NAMI California (CA), NAMI local affiliates (Mt. San Jacinto and San Bernardino), and the Roman Catholic Diocese of San Bernardino, who were actively involved in the study design and procedures (e.g., data collection materials, intervention development and delivery), data analysis and interpretation, and dissemination of study findings.

### Trial Design

A total of 14 parishes were enrolled before randomization (7 intervention; 7 wait-list control). Parishes [clusters] were matched in pairs based on size and number of Spanish language masses offered on Sundays (minimum of two) and randomly assigned within each pair to intervention or control. At least one member each from the Diocese of San Bernardino, a NAMI local affiliate, and RAND met and presented the study to priests and leaders from eligible parishes. Only one of the parishes originally approached declined participation given that an interim priest was in place at the time. Baseline and 12-month follow-up surveys were administered. Intervention activities followed the administration of baseline survey, while wait-list parishes were offered intervention activities after the 12-month surveys. All study procedures were approved by the institutional review board at RAND .

### Participants and Data Collection

Participants were informed about the study during Sunday service announcements or group ministry meetings and invited to English- and Spanish-language group self-administered survey sessions at parish sites before and after religious services or meetings. Informed consent was reviewed and obtained individually or in groups. Individuals who were ages 18 years or older, English or Spanish-speaking, and reported attending parish activities (e.g., mass, ministry meetings) at least two times per month were eligible to participate in the study. Participants could complete the survey in-person during on-site survey sessions or online. Options to complete the survey by mail or phone were added for 12-month surveys. Gift cards were provided for completing the baseline ($20) and 12-month ($30) surveys.

**Figure 1:**
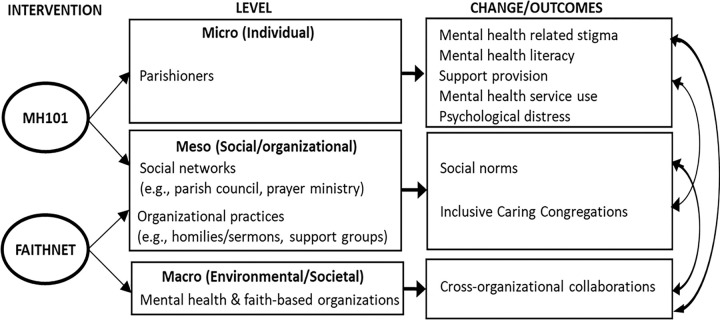
AMEN Conceptual Framework

A total of 1,715 participants completed the baseline survey across the 7 intervention (N=911) and 7 wait-list control parishes (N=804). Thirty-two percent (N=551) were lost to follow-up due to inability to contact (N=429), invalid contact information (N=63), illness (N=2), or refusal (N=57). See Figure 1 for participant flow diagram.

### Intervention

**Figure 2:**
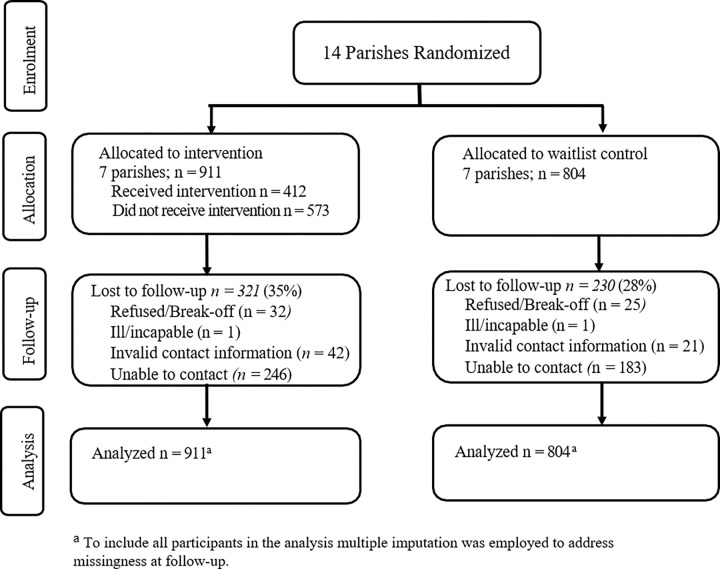
Cluster Randomized Controlled Trial Participant Flow Diagram

Based on conceptual frameworks for the Framework Integrating Normative Influences on Stigma [29] and mental health services disparities [30], the AMEN parish-based multi-level intervention targets mechanisms of disparities and stigma that occur at the individual, social/organizational, and environmental/societal levels (see Figure 2). The AMEN intervention included two NAMI programs, *Mental Health (MH)101* and *FaithNet*, both feature contact-based education, which has been shown to yield longer-term reductions in stigma [17]. MH101 is a 60–90 minute program that includes a psychoeducational presentation (on common types of mental illness, treatment, and stigma), a videoand live presenter stories of personal recovery and family member perspectives, and an interactive question and answer period. MH101 presenters provide information about NAMI resources and facilitate referrals to local mental health services. NAMI CA created MH101 as part of their efforts to develop culturally responsive, community driven mental health awareness programming that builds the capacity of those impacted by mental illness to give voice to their stories within the context of their own culture and community. NAMI CA conducted focus groups throughout the state of California with Hispanic individuals and families affected by mental illness. The main themes and recommendations from the focus groups informed the development of MH101; this included incorporating two presenters (an individual in recovery and a supporting family member or loved one) and having a Spanish language version available. MH101 was delivered to parishioners (*individual level*) and ministry groups (*social level*).

FaithNet includes Bridges of Hope (a contact-based educational presentation about mental illness, the role of the faith community in helping people and families affected by mental illness and NAMI resources), which was delivered to parish leadership to target *organization level* practices. In addition to contact-based education, FaithNet also contains resources such as information on how congregations can be inclusive and welcoming toward people with mental illness, tips on how to help a person with mental illness, and example services, sermons, prayers, and faith-based support groups that can help shape organizational practices. Parish pastors were asked to deliver at least one homily addressing mental illness stigma and treatment during mass. Research staff supplemented FaithNet resources by developing and providing parish pastors with a mental health information resource guide which contained information about the prevalence of mental illness, treatment, barriers to mental health services, and additional faith-based resources addressing mental illness (e.g., *Hope and Healing: A Pastoral Letter from the Bishops of California on Caring for Those who Suffer from Mental Illness Addressed to All Catholics and People of Goodwill* [31]; *American Psychiatric Association Foundation*: *Mental Health: A Guide for Faith Leaders* [32]. In addition, NAMI and research staff sent text messages regarding NAMI programs and mental health resources that were being offered. Parish pastors were also asked to implement at least two additional organizational practices to foster caring congregations (e.g. support groups). At the *environmental/societal level*, NAMI affiliates often have strong partnerships with local community-based mental organizations and serve as liaisons to mental health services.

After baseline surveys, NAMI programs were offered at a subsequent time to intervention parishes through a variety of venues (e.g., after masses, during ministry meetings). NAMI programs were delivered by two NAMI local affiliates, Mt. San Jacinto and San Bernardino. All NAMI facilitators have lived experience, identify as Hispanic and are from the local community, which allow for the delivery of contact-based, peer-delivered, culturally and linguistically sensitive programs. At each intervention parish, NAMI delivered at least one Bridges of Hope presentation and between three to seven MH101 presentations. Attendance sign-ins, independent fidelity ratings of MH101, and observations of homilies delivered by parish pastors were conducted to assess implementation. Comprehensive results of the process evaluation are reported separately.

### Measures

We evaluated the effect of a multi-level parish-based intervention on the following outcomes: mental health literacy, stigma, support for individuals with mental health problems, and mental health service use. *Demographic information* was collected and included as covariates (e.g., age, gender, marital status, education, nativity status, English/Spanish language proficiency, employment, income, health insurance status, and ease of getting medical care).

To capture mental health need, the Kessler-6, a six-item brief screener designed to measure non-specific psychological distress in diverse settings and populations was used. A total score of 13 or greater is indicative of *serious psychological distress* (i.e., a high likelihood of a clinical mental disorder warranting treatment) (1=Yes; 0=No). [33].

*Mental health literacy* was assessed with a 5-item mental health literacy scale developed for the lay community [34]. Respondents were asked to rate their level of agreement with each item (e.g., “Early diagnosis of a mental illness can improve chances of getting better”) from a scale of 1 (*strongly agree*) to 5 (*strongly disagree).*

Stigma was assessed across three dimensions. *Social distance* is one of the most commonly assessed dimensions of stigma and reflects a desire to avoid contact with people with mental illness [35]. Social distance was measured using the Reported and Intended Behavioral Scale which assesses respondents’ willingness to interact with someone with a mental health problem across four different circumstances. For example, respondents were asked to indicate how much they agree or disagree with the statement “In the future, I would be willing to live with someone with a mental health problem” using a scale of 1 (*strongly agree*) to 5 (*strongly disagree*)[36]. To assess the extent to which respondents anticipate being devalued by others in the community for having a mental illness, the *anticipated stigma* subscale of the Endorsed and Anticipated Stigma Inventory was used [37]. Respondents were asked to indicate their level of agreement (1=*strongly agree*; 5=*strongly disagree*) with four statements that began with the item stem, “If I had a mental health problem and friends and family knew about it, they would…think less of me; see me as weak; feel uncomfortable around me; think I was faking.” To assess stigma associated with treatment, the *Stigma Concerns about Mental Health Care* scale was modified to measure concerns about treatment for “a mental health problem” instead of for “depression.” Respondents were asked to rate on a scale of 1 (*strongly agree*) to 5 (*strongly disagree*) their agreement with four statements (e.g., “I would not want to receive treatment for a mental health problem because of being embarrassed to talk about personal matters with others”)[16]. Mean scores were derived with higher scores indicating greater mental literacy or stigma.

*Support provision* was assessed by asking in the past six months whether respondents had taken the following actions: “Spend time listening to someone about their mental health problem;” “Talk to someone about their suicidal thoughts;” Recommend to someone with a mental health problem to seek professional help;” “Give someone information about their mental health problem;” and “Give someone information about local mental health services”[12]. “Yes” responses were totaled to derive a mean sum score.

*Mental health service use* was measured by asking respondents if they had seen a primary care physician/general practitioner or any other professional, such as counselor, psychiatrist, or social worker for problems for problems with mental health, emotions, nerves, or use of alcohol or drugs (1=Yes; 0=No) [38].

Measures that were only available in English (i.e., mental health literacy, social distance, and anticipated stigma) were translated into Spanish by a certified translator and then two bilingual research team members with related content expertise reviewed the translation and identified and reconciled discrepancies to ensure the translation was contextually appropriate for the study population [39].

### Statistical Analysis

We conducted a power analysis prior to data collection using R 4.0, planning for 1,400 participants over two waves of repeated measurements, with a power >.80 and two-sided p-value <.05. In a difference-in-differences (DID) comparison, the detectable effect sizes ranged from .249 to .501 on Cohen’s d. Additional details can be found elsewhere [40].

Data analyses were conducted with Statistical Analysis System (SAS) 9.3. We first performed 20 rounds of multiple imputation to impute the few missing entries in baseline covariates and missing outcomes at 12-month follow-up. Next, we conducted intent-to-treat (ITT) analysis using the original randomization status. For numerical outcomes, we fitted the DID longitudinal regression using the study arm, wave, and their two-way interactions, while adjusting for baseline covariates. The coefficient of the two-way interaction term represented the treatment effect after accounting for secular trends and baseline differences. Two random effects were employed to account for serial correlations within the same participant and intra-class correlations among participants in the same parish. For binary outcomes (i.e., serious psychological distress, mental health service use), we used repeated-measure logistic regression by the generalized estimating equation with the same setting as the models for numerical outcomes. The coefficient of the two-way interaction term represented the adjusted treatment effect. Effect estimates were converted to Cohen’s d. Lastly, we conducted as-treated analysis by excluding those in the treatment arm but reporting having not participated in any treatment activity. Except for the subsample, the as-treated analysis applied the same modeling approach as in the ITT analysis.

## RESULTS

Participant characteristics are presented in Table 1. Of the total sample of 1,715 participants, the mean age was 48 years old (SD = 14.4) and the majority identified as female (73%; N = 1,252), married or living with a partner (69%; N = 1,183), having completed a high school education or less (68%; N = 1,166), and opted to complete the surveys in Spanish (82%; N = 1,406). Nine percent of participants (N = 154) met criteria for serious psychological distress and nearly a fifth (19%; N = 326) had received mental health services in the past 12 months. Fourteen parishes were cluster randomized to either the intervention (N = 911) or control (N = 804) condition. Table 1 shows that there were no significant demographic differences between the two study conditions with the exception of language proficiency (p < .05) and Hispanic subgroup (p < .05). Intervention versus control condition participants were more likely to be bilingual (38% vs. 33%) and identify as Mexican (89% vs. 84%) and less likely to identify as Central American (4% vs. 9%).

Of the 911 participants in the intervention parishes, only 45% (N = 412) reported participating in at least one AMEN intervention activity (see “as-treated” intervention group in Table 1). Intervention vs. control participants within the “as-treated” group exhibited no significant demographic differences except for language proficiency (p < .05) and Mexican (p < 05) and Central American (p < .05) subgroup identification.

As seen in Table 2, ITT analyses revealed that participants in intervention parishes exhibited no significant differences with respect to mental health literacy (p = .12), social distance (p = 0.48), anticipated stigma (p = 0.55), mental health care stigma (p = 0.64), support provision (p = 0.47), mental health service use (p = 0.24), or serious psychological distress (p = 0.59) compared to participants in control parishes. However, as-treated analyses indicated that the subset of participants who reported engaging in the intervention demonstrated significant decreases in social distance (β = −0.15; p < .01), significant increases in support provision (β = 0.32; p < .05), and significant decreases in serious psychological distress (OR = 0.45; p < .01) compared to participants in control parishes.

## DISCUSSION

This is the first RCT to address U.S. Hispanic mental health disparities within a faith-based congregational setting using a multi-level, multi-component intervention to improve mental health literacy, stigma, support for individuals with mental illness, mental health service use, and psychological distress. Although ITT analyses did not detect significant overall intervention effects, “as-treated” analyses indicated potentially promising results among intervention parish participants who reported engaging in at least one AMEN activity. Compared to participants in control parishes, participants within intervention parishes who had been exposed to the intervention exhibited significant decreases in social distance and improvements in support provided to individuals with a mental illness and in rates of serious psychological distress. Given that social distance has been shown to be one of the most obdurate domains of stigma that is resistant to change [41, 9], this study’s findings are encouraging and consistent with prior meta-analyses that have shown contact-based education to be effective in reducing social distance [42, 43]. However, evidence for the effectiveness of contact-based education in sustaining longer-term effects is limited and little attention has been paid to how aspects of identity such as race and ethnicity may impact outcomes [17, 44]. This study provides preliminary evidence that contact-based education programs may be able to yield longer-term reductions in stigma among Hispanic populations and builds on findings from a statewide mental illness stigma reduction initiative involving the dissemination of contact-based education programs throughout California in which Hispanic participants exhibited significantly greater pre-post decreases in stigma (including social distance) compared to non-Hispanic White participants [44].

The fact that intervention parish participants who had engaged in AMEN activities provided significantly more support to individuals with a mental illness relative to control parish participants is noteworthy given that a systematic meta-review of meta-analyses indicated that contact-based education programs did not yield significant effects on behavior outcomes, neither in the short- or longer-term follow-up periods [17]. The impact of AMEN on behavioral outcomes may have been due to the nature of it being a multicomponent, multilevel stigma reduction intervention which is rare but thought to have more far-reaching effects than single-level interventions [17, 45]. Further, the significantly greater decreases in rates of psychological distress observed among intervention parish participants exposed to AMEN activities compared to control parish participants highlight the importance of understanding the impact of stigma interventions disseminated to the broad public among individuals with mental illness. An RCT involving Mental Health First Aid training delivered in a workplace setting also found significant intervention effects for improvements in mental health [46]. Reducing public stigma may indirectly improve mental health by reducing social isolation and discrimination [47] particularly when multilevel interventions are implemented within an organization or community setting, suggesting further research on the potential therapeutic effects of stigma interventions are warranted.

The lack of significant AMEN intervention effects on mental health literacy and service use while disappointing is not unexpected. Generally, contact-based education programs do not focus on mental health literacy outcomes but rather on stigma reduction, in contrast to mental health literacy programs that specifically target mental health knowledge and show improvements in this domain [17]. Additionally, there is limited evidence that stigma interventions can lead to long-term increases in mental health service use [48]. However, several RCTs employing either a fotonovela (photo comic booklets) [49, 50] or telenovela (television soap opera in Hispanic communities) [51] demonstrated significant short-term improvements in mental health literacy among Hispanic adults in community settings. Further, a RCT of a brief video intervention containing people sharing their personal experiences with depression showed significant long-term increases in mental health service use [52]. Future research could explore whether incorporating indirect contact-based education via fotonovelas, telenovelas, and brief videos of individuals sharing about their personal recovery as part of a multilevel, multicomponent parish-based intervention like AMEN could improve and sustain gains in mental health literacy and service use.

## Limitations

Although the study achieved an acceptable follow-up survey response rate, findings may be subject to non-response bias. This study’s analyses controlled for nativity status and language proficiency, but intervention effects could vary by these indices of acculturation and should be explored in future analyses. Participants primarily comprised individuals of Mexican descent and small numbers of other Hispanic subgroups precluding an examination of potential subgroup differences. The intervention was conducted within the Roman Catholic Church, which is the largest religious group among Hispanics in the U.S. [53], but findings may not generalize to other religious traditions. The follow-up survey was intended to be deployed six months after the baseline survey, but the scheduling of the intervention activities and follow-up survey were delayed because of competing parish priorities and demands. Thus, the study was unable to capture shorter-term intervention effects that may have transpired.

Only 45% of intervention parish participants attended at least one AMEN activity. This may have been due in part to the pragmatic nature of the study. Participants were provided opportunities to complete the baseline survey after Sunday masses or during parish gatherings and NAMI programs were then offered at subsequent times to the entire parish. In contrast, most stigma intervention studies typically ask participants to consent to taking a baseline survey that is immediately followed by the intervention which virtually ensures 100% intervention exposure. As treated analyses suggest that intervention effects may have been stronger if all intervention parish participants were exposed to the AMEN program. Additional study is needed to understand barriers to engaging in parish-based mental health activities and effective strategies for more fully involving parishioners (e.g., integrating mental health education as part of parish life).

A recent review of partnerships between faith communities and the mental health sector [54] highlighted that when church volunteers took on greater roles – delivering services across the continuum of care (e.g., psychoeducation, referrals and linkage to mental health services) – greater engagement ensued (e.g., 95% of individuals who were referred used services) [55]. Faith community members often share similar cultural backgrounds which may facilitate better understanding, communication, and trust as evidenced in culturally adapted mental health interventions and peer support models that have shown improvements in treatment adherence and outcomes when providers with shared experiences deliver services [56, 57]. Thus, training faith community members to deliver NAMI programs that provide psychoeducation (e.g., MH101), family support (Family to Family), and peer support (Peer-to-Peer) as well as Mental Health First Aid (referral and linkage to mental health services) may be a means to bolstering engagement and efforts to more effectively reduce stigma and unmet mental health needs.

## CONCLUSIONS

This study demonstrated that when exposed to a multilevel, multicomponent stigma intervention within a faith-based congregational setting positive outcomes were observed at a notably longer-term follow up period. This is noteworthy given the limited evidence for the long-term effectiveness of stigma interventions which has been attributed to the rarity of multilevel, multicomponent stigma interventions [17]. Continuous, repeated, and longer contact interventions have also been posited as necessary to invoke enduring changes in stigma [58, 43]. Multilevel, multicomponent, continuous stigma interventions are scant likely because they are complex, resource intensive, and difficult to maintain [48, 59]. With the exception of programs delivered by NAMI [60, 61] and Mental Health First Aid [62], it is unclear the extent to which stigma interventions that have been studied and tested are still being delivered and sustained. Partnerships in which faith communities serve as co-leaders and implementers of a comprehensive set of mental health education and support programs that are offered up as part of regular and continual care for their members and community may be the type of robust and sustainable approach that can yield long lasting reductions in stigma and significant impacts on access and engagement with needed mental health services.

## Figures and Tables

**Table 1:** Baseline characteristics of participants

			Intent to treat					As-Treated				
	Total (N = 1715)		Intervention (N = 911)		Control (N = 804)			Intervention (N = 412)		Control (N = 804)		
Characteristics	N or Mean	% or SD	N or Mean	% or SD	N or Mean	% or SD	p	N or Mean	% or SD	N or Mean	% or SD	p
**Demographic variables** ^a^												
Age in years (M ± SD)	48.0	14.4	47.4	14.9	48.7	13.7	0.06	47.5	13.9	48.7	13.7	0.14
Female	1252	73%	672	74%	580	72%	0.18	314	76%	580	72%	0.19
Marital status^a^							0.38					0.68
Single	273	16%	157	17%	116	14%		66	16%	116	14%	
Married/living with partner	1175	69%	613	67%	562	70%		280	68%	562	70%	
Divorced/Separated/Widowed	231	13%	120	13%	111	14%		61	15%	111	14%	
Education^a^							0.69					0.07
Less than high school	722	42%	391	43%	331	41%		179	43%	331	41%	
High school graduate or GED	452	26%	235	26%	217	27%		101	25%	217	27%	
Associate’s degree or some college	296	17%	162	18%	134	17%		83	20%	134	17%	
Bachelor’s degree	143	8%	69	8%	74	9%		26	6%	74	9%	
Graduate degree	58	3%	33	4%	25	3%		18	4%	25	3%	
Completed survey in Spanish	1398	82%	748	82%	650	81%	0.50	344	84%	650	81%	0.26
Language proficiency							**0.01**					**0.01**
Bilingual	615	36%	348	38%	267	33%		163	40%	267	33%	
English	44	3%	18	2%	26	3%		5	1%	26	3%	
Spanish	978	57%	513	56%	465	58%		233	57%	465	58%	
Neither	50	3%	17	2%	33	4%		8	2%	33	4%	
Nativity Status (U.S. Born)	280	16%	153	17%	127	16%	0.33	70	17%	127	16%	0.77
Ethnicity^b^												
Mexican	1485	87%	808	89%	677	84%	**0.01**	366	89%	677	84%	**0.03**
Central American	104	6%	35	4%	69	9%	**<.001**	21	5%	69	9%	**0.03**
Other	29	2%	15	2%	14	2%	0.88	9	2%	14	2%	0.59
**Outcome variables**												
Mental health literacy	3.4	0.8	3.4	0.8	3.5	0.8	0.32	3.5	0.8	3.5	0.8	0.54
Stigma												
Social distance	2.4	1.1	2.4	1.1	2.5	1.1	0.41	2.4	1.1	2.5	1.1	0.22
Anticipated stigma	2.3	1.2	2.4	1.2	2.3	1.2	0.61	2.3	1.2	2.3	1.2	0.82
Mental health care stigma	1.8	0.8	1.8	0.8	1.8	0.8	0.58	1.7	0.8	1.8	0.8	0.41
Support provision	1.8	1.8	1.9	1.8	1.7	1.8	0.08	2.1	1.8	1.7	1.8	0.002
Serious psychological distress	160	9%	97	11%	63	8%	0.11	50	12%	63	8%	0.05
Past 12 month mental health service use	325	19%	181	20%	144	18%	0.29	88	21%	144	18%	0.34

a Categories do not sum to total sample because of missingness for sex (n = 26), marital status (n = 36), education (n = 44), nativity status (n = 41), ethnicity (n = 41).

b Groups not mutually exclusive.

**Table 2:** Intent to Treat and As Treated Analyses on the Effects of a Parish-Based Intervention^a^

	Intent to Treat (N = 1715)				As-Treated (N = 1216)			
	β	95% CI	p	Effect size	β	95% CI	p	Effect size
Mental health literacy	0.08	−0.02–0.18	0.12	0.10	0.09	−0.02–0.20	0.12	0.11
Stigma								
Social distance	−0.07	−0.18–0.05	0.25	−0.06	−0.15	−0.27– −0.04	0.009	−0.14
Anticipated stigma	0.04	−0.10–0.18	0.56	0.03	−0.005	−0.14–0.13	0.95	−0.004
Mental health care stigma	−0.03	−0.11–0.06	0.55	−0.04	−0.05	−0.13–0.04	0.30	−0.06
Support provision	0.14	−0.08–037	0.21	0.08	0.32	0.10–0.54	0.005	0.18
	ORR	95% CI	p	Effect size	ORR	95% CI	p	Effect size
Serious psychological distress	0.84	0.52–1.36	0.47	−0.05	0.45	0.25–0.81	0.008	−0.17
Mental health service utilization	0.84	0.63–1.11	0.20	−0.08	0.97	0.71–1.31	0.82	−0.01

a Analyses adjusted for demographic covariates (i.e., age, gender, marital status, education, nativity status, English/Spanish language proficiency, employment, income, health insurance status, and ease of getting medical care).

a To include all participants in the analysis multiple imputation was employed to address missingness at follow-up.

## Footnotes

1. The Diocese of San Bernardino uses the term Hispanic when referring to their ministries and parish community which is the term adopted in this study per Publication Manual of the American Psychological Association.
